# Comparative Analysis of Tentacle Extract and Nematocyst Venom: Toxicity, Mechanism, and Potential Intervention in the Giant Jellyfish *Nemopilema nomurai*

**DOI:** 10.3390/md22080362

**Published:** 2024-08-09

**Authors:** Xiao-Yu Geng, Ming-Ke Wang, Xiao-Chuan Hou, Zeng-Fa Wang, Yi Wang, Die-Yu Zhang, Blessing Danso, Dun-Biao Wei, Zhao-Yong Shou, Liang Xiao, Ji-Shun Yang

**Affiliations:** 1 Naval Medical Center of PLA, Naval Medical University, Shanghai 200052, China; 15339625597@163.com (X.-Y.G.); wmke021@163.com (M.-K.W.); 2 Faculty of Naval Medicine, Naval Medical University, Shanghai 200433, China; xiaochuanhou@126.com (X.-C.H.); wangzengfa2022@126.com (Z.-F.W.); wyi0815@hotmail.com (Y.W.); blessingdanso@hotmail.com (B.D.); 3 College of Traditional Chinese Medicine, Jilin Agricultural University, Changchun 130118, China; 4 College of Pharmacy, Bengbu Medical University, Bengbu 233030, China; 4323@outlook.com; 5 Unit 92196 of the People’s Liberation Army, Qingdao 266000, China; wei1026627857@163.com; 6 Faculty of Health Service, Naval Medical University, Shanghai 200433, China

**Keywords:** jellyfish venom, toxic effects, extraction, PLA2, verapamil, EDTA, PACOCF3

## Abstract

The giant jellyfish *Nemopilema nomurai* sting can cause local and systemic reactions; however, comparative analysis of the tentacle extract (TE) and nematocyst venom extract (NV), and its toxicity, mechanism, and potential intervention are still limited. This study compared venom from TE and NV for their composition, toxicity, and efficacy in vitro and in vivo used RAW264.7 cells and ICR mice. A total of 239 and 225 toxin proteins were identified in TE and NV by proteomics, respectively. Pathological analysis revealed that TE and NV caused heart and liver damage through apoptosis, necrosis, and inflammation, while TE exhibited higher toxicity ex vivo and in vivo. Biochemical markers indicated TE and NV elevated creatine kinase, lactatedehydrogenase, and aspartate aminotransferase, with the TE group showing a more significant increase. Transcriptomics and Western blotting indicated both venoms increased cytokines expression and MAPK signaling pathways. Additionally, 1 mg/kg PACOCF3 (the phospholipase A2 inhibitor) improved survival from 16.7% to 75% in mice. Our results indicate that different extraction methods impact venom activities, tentacle autolysis preserves toxin proteins and their toxicity, and PACOCF3 is a potential antidote, which establishes a good extraction method of jellyfish venom, expands our understanding of jellyfish toxicity, mechanism, and provides a promising intervention.

## 1. Introduction

Venoms are usually defense mechanisms or hunting weapons evolved from various biological metabolites, and their compositions are very complex, including proteins, peptides, and small molecules [1]. Due to their potent biological activities and in-depth research, venoms as therapeutic drug candidates have gradually become a hot spot worldwide [2,3,4,5,6]. Marine organisms have always been an excellent source of innovative compounds and have great potential in the development of new medicines, but compared with terrestrial venom, research progress has been slow. The main limitations include the difficulty of venom extraction, the activity of which is heavily affected by the seasons, the lack of information about the venom, and so on. Establishing a method of venom extraction that maintains their activities completely is an essential foundation for the research and development of the venom [7,8].

The cnidarians are an essential component of the marine ecosystem, with about 3900 described species, mainly comprising the Anthozoa, Endocnidozoa, and Medusozoa divisions. The Medusozoa is one of the most diverse and complex taxa in the cnidarian phylum [9], and is broadly divided into four distinct lineages, including Hydrozoa, Acraspeda, Cubozoa, and Scyphozoa [10,11]. Scyphozoa is one of the most recognizable species of Medusozoa, which is widely distributed and highly toxic [12]. Meanwhile, increases in Scyphozoa jellyfish abundance have been found in some sea areas, such as the eastern Atlantic and the western Pacific [13,14,15], with significant ecological, economic, and human health impacts [16,17,18,19]. The *Nemopilema nomurai* (*N. nomurai*) is one of the most toxic Scyphozoa jellyfish and the main poisonous jellyfish species in China, Japan, and Korea [16,20]. The toxin components of jellyfish venom are complex and have evolved, and their compositions are diverse among species [21,22]. However, in general, jellyfish venom is usually composed of proteins and peptides used by jellyfish for predation or defense [23]. Jellyfish toxins can cause various biological effects, including hemolytic toxicity [24], cardiovascular toxicity [25], neurotoxicity [26], and skin [27] and muscle toxicity [28]. Depending on the species, jellyfish stings can induce effects ranging from localized itching and erythema to systemic shock, pulmonary edema, and even death [29,30,31,32].

Several proteins have been identified or characterized from *N. nomurai* sting venom by traditional methods, such as multilayer chromatography [25], sequential chromatography [33], matrix-assisted laser desorption/ionization time-of-flight mass spectrometry (MALDI-TOF MS) [23], gene cloning, and expression methods [34]; however, the results obtained are still limited. Recently, the emergence of high-throughput technologies combined with bioinformatics has dramatically facilitated the study of venom protein/peptide diversity in a variety of animals, which often integrates genomics, transcriptomics, and proteomics [35,36]. Recently, a tissue proteomic analysis of *N.nomurai* sting venom-induced dermatitis model was published [37], which benefited from the sequencing and annotation of the *N. nomurai* sting jellyfish proteome [20]. Proteomic methods identified metalloproteinases, proteases, and pore-forming toxins in *N.nomurai* sting venoms. Later, Li’s research isolated the lethal components of *N. nomurai* sting toxins [25]. Proteomic analysis identified 13 toxin homologs, including phospholipases, potassium channel inhibitors, hemolysins, and thrombin. Yu et al. [37] also identified zinc metalloproteinases using liquid chromatograph mass spectrometer (LC-MS/MS) fractions, serine proteases, and so on. However, most of them do not provide absolute evidence that all the identified proteins are venom components, and there are slight differences in the screening criteria between these individuals, such as different fold changes in differentially expressed genes (DEGs), and some different e-values may be controversial [38]. Several transcriptomic studies have helped us to better understand the evolutionary relationships [39,40] and the mechanisms [41] by which the toxin exerts its effects. The results of these studies are discrepant [8], and the relevant genomic data still need to be improved. Therefore, there is an urgent need to explore the venom components and their effect mechanisms by applying various research techniques at the molecular level and conducting extensive analyses to accelerate the development of therapeutic drugs and the process of venom transformation.

Venom collection has been the basis for the evolution of venomous organisms [42], toxic effects, and the development of immunopharmaceuticals [43]. Relatively standardized venom collection methods have been established for snakes, scorpions, spiders, and bees [42,44,45,46,47]. This has facilitated the process of their toxic studies to a certain extent. Some studies have shown that different methods of venom extraction may affect their composition or distribution of toxin proteins in the venom [47,48]. Venom extraction from nematocysts is a prerequisite for toxicity studies of cnidarians. The currently used methods of jellyfish venom collection can be broadly categorized into two types, both of which require low-temperature autolysis of the collected jellyfish tentacles, and the difference lies in whether or not they are homogenized and the form of dialysis that follows [7,41,49,50]. We distinguished between the extraction methods of tentacle extraction (TE) and nematocyst venom extraction (NV) [51] based on whether the homogenization was performed. Unfortunately, there is no systematic comparison of the effects of different extraction methods on the venom composition, nor is there a functional assay comparing the 50% lethal dose (LD50) to determine the optimal purification method [7]. Obtaining sufficient venom without destroying the toxin proteins is of great value in fully utilizing jellyfish resources and improving the efficiency of jellyfish venom research. In addition to venom collection methods, the effective inhibitors are also controversial [30,52], and the use of inappropriate inhibitors or drugs may even worsen the clinical course of jellyfish stings [53].

In this study, we constructed an ex vivo and in vivo jellyfish toxicity model using RAW264.7 cells and ICR mice, which were exposed to naturally dissolved and mechanically crushed extracted jellyfish venom, and then compared their toxin compositions and ex vivo and in vivo toxicity effects by combining this model with proteomics analyses, histological analyses, and biochemical results. In addition, the inhibitory effects of the phospholipase A2 (PLA2) inhibitor PACOCF3, the matrix metalloproteinase (MMP) inhibitor EDTA, and the Ca^2+^ channel inhibitor verapamil on the toxicity of jellyfish venom were evaluated in vitro and in vivo. For the first time, our study compares the venom extracted from jellyfish by different methods, which is not only conducive to the establishment of a more stable and efficient method of venom extraction but also of great significance for improving the jellyfish toxin library and developing potential antidotes.

## 2. Results

### 2.1. Comparison of Toxins in TE and NV and Toxicity Evaluation

**Figure 1:**
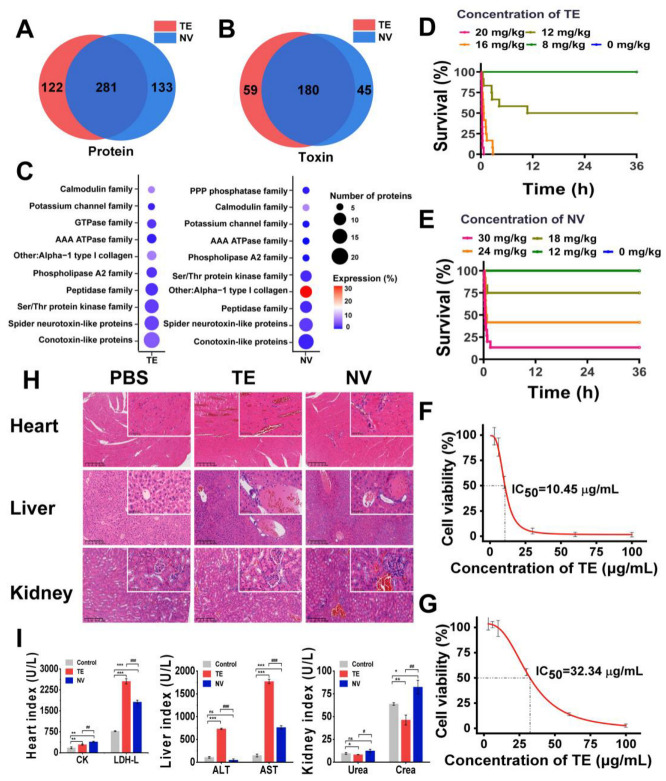
Comparison and toxicity evaluation of toxins in TE and NV. (**A**) Venn analysis of proteins between TE and NV groups, n = 4. (**B**) Venn analysis of toxins between TE and NV groups, n = 4. (**C**) Overview of toxin family distributions and protein number and expression in TE and NV identified in the proteomes. (**D**) Survival analysis of ICR mice after TE injections, n = 12. (**E**) Survival analysis of ICR mice after NV injections, n = 12. (**F**) Viability of RAW264.7 cells exposed to TE, n = 6. (**G**) Viability of RAW264.7 cells exposed to NV, n = 6. (**H**) HE analysis of the heart, liver, and kidneys at 400× magnification, n = 4. (**I**) Evaluation of blood biochemical indices, including creatine kinase (CK) and lactate dehydrogenase (LDH), aspartate aminotransferase (AST), alanine aminotransferase (ALT), creatinine (CREA), and urea nitrogen (UREA), n = 3. * *p* < 0.05, ** *p* < 0.01, and *** *p* < 0.001 represent the comparison of the group with the control group, and # *p* < 0.05, ## *p* < 0.01, and ### *p* < 0.001 represents the comparison of the group with the TE group, with ns representing no significance.

Based on label-free proteomics analysis utilizing LC-MS/MS, we conducted a comprehensive investigation of the compositions of TE and NV. By consulting the cnidarian protein database, we identified a total of 403 proteins in TE and 414 proteins in NV, with 281 proteins common to both groups (Figure 1A). Given the limited availability of toxin data within the cnidarian database, we employed BLASTp [54] to run the UniProt toxin database (Tox-Prot) in conjunction with the *N. nomurai* transcriptome database previously established by our research team. We derived the predicted toxin sequences from the *N. nomurai* transcriptome with the filtering criteria, including e-value < 1.0 × 10^−p^ and %identity > 40%. Utilizing the inferred toxin data as a reference point, we successfully identified 239 and 225 predicted toxin proteins in TE and NV, respectively (Figure 1B). The toxin characteristics of both venoms exhibited notable similarities. We classified these toxins into ten distinct families based on the expression levels and quantities of the corresponding toxin proteins, which included conotoxin-like proteins, spider neurotoxin-like proteins, Ser/Thr protein kinase, peptidase, phospholipase A2, AAA ATPase, calmodulin, PPP phosphatase, GTPase, and other toxins. It is important to note, however, that the number and relative expression levels of proteins within these toxin families may differ (Figure 1C).

First, we constructed an in vivo *N. nomurai* sting model by injecting TE into the tail vein of ICR mice at 4–6 h intervals and found that TE in the range of 12–20 mg/kg was potentially lethal, while NV in the range of 18–30 mg/kg was potentially lethal (Figure 1D,E). Quantitative analysis showed that the LD50 was 12.19 mg/kg for TE and 22.52 mg/kg for NV (Figure S1A,B). Both TE (>16 mg/kg) and NV (>18 mg/kg) mice showed an accelerated heart rate, shortness of breath, and muscle weakness at high doses, except that the TE mice showed more convulsions, which were not observed in the NV group. Pathological analysis showed that TE and NV caused heart and liver damage in the ICR mice. There was structural damage and severe congestion of cardiomyocytes, deformation of liver lobules, and necrosis of hepatocytes, accompanied by localized inflammatory cell infiltration, while the damage to renal tissues was less pronounced in the TE group. More severe damage to the cardiomyocytes was observed in the NV group, with cellular arrangement disorganization, more extensive hepatocytes necrosis, and severe lesions of glomeruli with a sizeable inflammatory infiltrate (Figure 1H). Moreover, biochemical evaluation showed that the cardiac markers creatine kinase (CK) and lactate dehydrogenase (LDH-L) were significantly elevated after both TE and NV exposures. The hepatic markers aspartate aminotransferase (AST) and alanine aminotransferase (ALT) were increased in vivo in the TE group, and even though the ALT in the NV group did not show any abnormality, its elevated AST was equally suggestive of severe liver injury, while creatinine (CREA) was also upregulated in the NV group (Figure 1I). In addition, in vitro modeling experiments also illustrated the toxicity differences between TE and NV. Cell viability decreased in both TE and NV groups, but still showed differences, with IC_50_ of 10.45 μg/mL (Figure 1F) and 32.34 μg/mL (Figure 1G), respectively.

### 2.2. TE and NV Lead to the Upregulation of the Inflammatory Signaling Pathway and the Release of Inflammatory Factors

**Figure 2:**
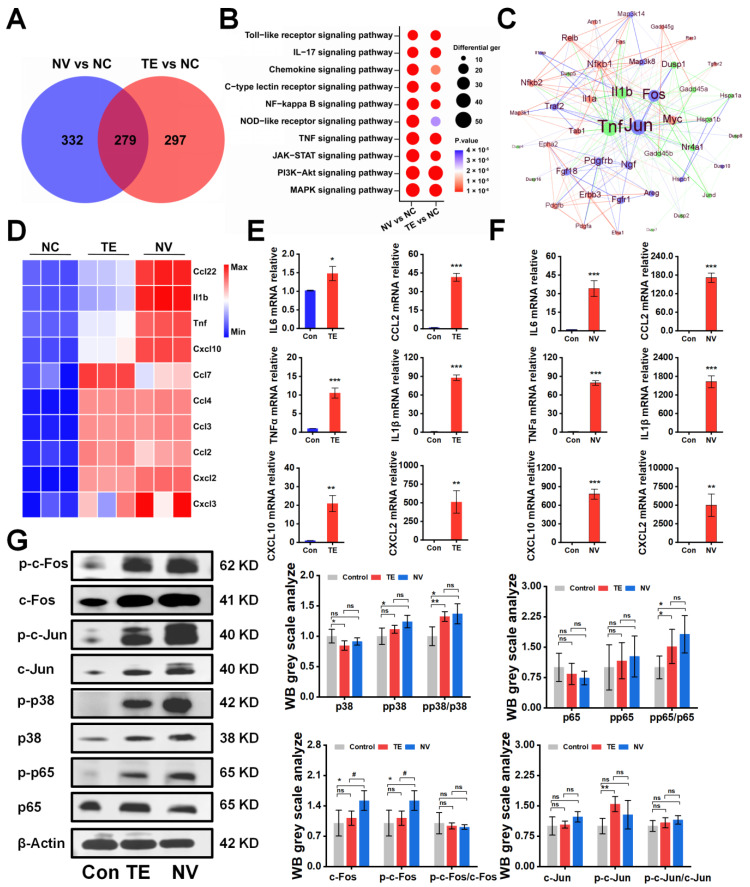
TE versus NV transcriptome overview and Western blot results. (**A**) Venn diagram of genes upregulated by TE versus NV in RAW264.7 compared to PBS treatment, n = 4. (**B**) Bubble plots of KEGG enrichment analysis of DEGs. (**C**) Protein–protein interaction networks of TE (10.45 μg/mL) and NV (32.34 μg/mL). (**D**) Comparative analysis of TE and NV co-induced upregulation of inflammatory factors in the transcriptome. All genes were calculated using log2 (fold change) and shown by thermography. (**E**,**F**) Relative gene expression of six inflammatory factors including IL-6, TNF-α, Cxcl10, IL1β, Ccl2, and Cxcl2 in RAW264.7 cells treated with TE (10.45 μg/mL) or TE (32.34 μg/mL), n = 3. (**G**) Western blotting analysis of c-fos, c-Jun, p65, and p38 phosphorylated and non-phosphorylated protein, n = 4. * *p* < 0.05, ** *p* < 0.01, and *** *p* < 0.001 represent the comparison of the group with the control group, and # *p* < 0.05 represents the comparison of the group with the TE group, with ns representing no significance.

To investigate the potential molecular mechanisms underlying the differences in the cytotoxicity of TE and NV treatments on RAW264.7, transcriptomic sequencing was performed, and differentially expressed genes (DEGs) were screened between the TE, NV, and control groups, respectively. PCA analysis showed significant differences in the transcriptomic profiles between the TE or NV groups and the control group (Figure S2A,B). Specifically, 1647 (576 upregulated and 1071 downregulated) genes were identified between the TE and control groups, and 1541 (611 upregulated and 930 downregulated) genes were identified between the NV and control groups (Figure S2). Venn diagram analysis showed that among the upregulated genes, 279 genes were common differential genes in the TE and NV groups as compared with the control group (Figure 2A). KEGG pathway analysis revealed similar enriched pathways in the TE group, including MAPK and PI3K-Akt, but the extent of gene enrichment varied. According to the degree of enrichment, the most highly enriched signaling pathways were MAPK and PI3K-Akt in the TE and NV groups, respectively (Figure 2B). It is noteworthy that although the PI3K-Akt pathway was highly enriched in the NV-treated group, the gene set enrichment analysis (GSEA) evaluation did not show statistical significance (Figure S2C,D).

Meanwhile, the screening of transcriptome upregulated genes suggested that TE and NV exposure resulted in elevated transcript levels of inflammatory factors in RAW264.7 cells. Compared with the control group, TE and NV jointly induced upregulation of transcript levels of factors, such as Ccl22, IL1β, and TNFα (Figure S2C,D). However, the induction degree might differ among the demonstrated jointly upregulated cytokines, except for Ccl7 (Figure S2C,D). NV appeared to regulate cytokines to a greater extent than TE (Figure 2D). RT-qPCR showed that the TE- and NV-treated RAW264.7 groups differentially increased the transcriptional expression levels of the cytokines IL6, TNFα, Cxcl10, Ccl2, IL1β, and Cxcl2 compared to the control group, while the stimulatory effect of NV on cellular inflammatory factors was more significant than that of TE (Figure 2E,F).

We extracted protein–protein interaction networks (PPIs) from the transcriptome database and visualized them to predict common differential genes’ interaction and adhesion pathways. The PPI network of common DEGs contained 49 nodes and 233 edges (Figure 2C). Most interconnected nodes were considered hub genes in the PPI network, which revealed that Jun, Fos, and others were the most influential genes in the TE-treated group. In contrast, the NV-treated group exhibited NF-κB, Relb, Myc, and others as crucial influencers alongside the standard hub. The common pivotal genes include essential cytokines, such as IL1β and TNF, and other immune-regulating factors, such as Dusp1 and Nr4a1 (Figure 2C), which may be potential biomarkers and provide new therapeutic strategies for studying stinging injuries.

The Western blot showed that TE and NV significantly induced the phosphorylation of p65 and p38 to a comparable extent in RAW264.7 cells stimulated with TE and NV, respectively, when analyzing their total proteins and phosphorylated c-Fos, c-Jun, p65, and p38 (Figure 2G). It is worth mentioning that although the phosphorylation levels of c-Fos and c-Jun did not increase, their total protein expression was significantly increased, which was consistent with the results suggested by PPI.

### 2.3. Effect of Inhibitors on TE or NV Cytotoxicity

**Figure 3:**
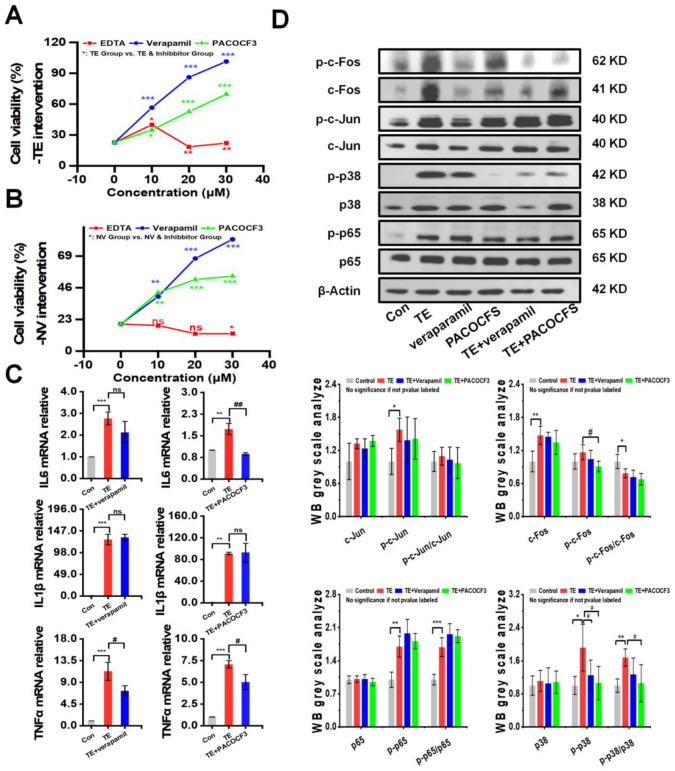
PACOCF3 and verapamil antagonized TE or NV toxicity in vitro. (**A**,**B**) Inhibition of TE or NV cytotoxicity in RAW264.7 cells by 10–30 μM EDTA, PACOCF3, and verapamil. n = 6. (**C**) Relative gene expression levels of three inflammatory factors, namely IL-6, IL1β, and TNF-α, in RAW264.7 cells treated with TE (10.45 μg/mL) or TE combined with PACOCF3 (20 μM) or verapamil (20 μM), n = 3. (**D**) Western blotting analysis of c-Fos, c-Jun, p65, and p38 phosphorylated and non-phosphorylated proteins, n = 4. * *p* < 0.05, ** *p* < 0.01, and *** *p* < 0.001 represent the comparison of the group with the control group, and # *p* < 0.05, and ## *p* < 0.01 represents the comparison of the group with the TE group, with ns representing no significance.

The intervention of TE or NV with different concentrations of inhibitors, including the MMP inhibitor EDTA, the PLA2 inhibitor PACOCF3, and the calcium channel inhibitor verapamil, showed that PACOCF3 and verapamil at 10–30 μM doses had strong antagonistic action against the cytotoxicity in the TE or NV treatments, while EDTA did not have a significant action (Figure 3A,B). Additionally, PLA2 and MMP activities showed a dependence on the TE or NV concentration. Therefore, it could be inferred that the negative efficacy of EDTA was independent of the MMP activity in the venom (Figure S3A,B). RT-qPCR results showed that most of the TE-stimulated rise in the inflammatory factors TNFα, IL6, and IL1β was not decreased by PACOCF3 or verapamil, and only the transcript levels of IL6 and TNFα were significantly inhibited by PACOCF3 and verapamil, respectively (Figure 3C). Notably, even in the absence of TE intervention, PACOCF3 and verapamil increased the transcriptional levels of IL6 in RAW264.7 cells, while verapamil mainly affected the transcriptional levels of IL1β (Figure S3C).

To investigate whether PACOCF3 and verapamil could reduce the expression of NF-κB and MAPKs signaling pathways, RAW264.7 was treated with TE dissolved in PBS alone or in combination with PACOCF3 and verapamil, and the results showed that PACOCF3 significantly inhibited TE-induced phosphorylation of p38, which was also observed in the presence of verapamil but did not show statistical significance. In addition, PACOCF3 and verapamil did not show modulation of the total protein or phosphorylation of c-Jun and p65. It should be noted that although PACOCF3 and verapamil did not show any regulation of c-Fos phosphorylation, the total protein level of c-Fos was significantly downregulated by these two inhibitors (Figure 3D). Moreover, the inhibitors themselves did not affect the protein, either in terms of total protein expression or their phosphorylation (Figure S3D).

### 2.4. Evaluation of Antagonistic Effects of PACOCF3 and Verapamil In Vivo

**Figure 4:**
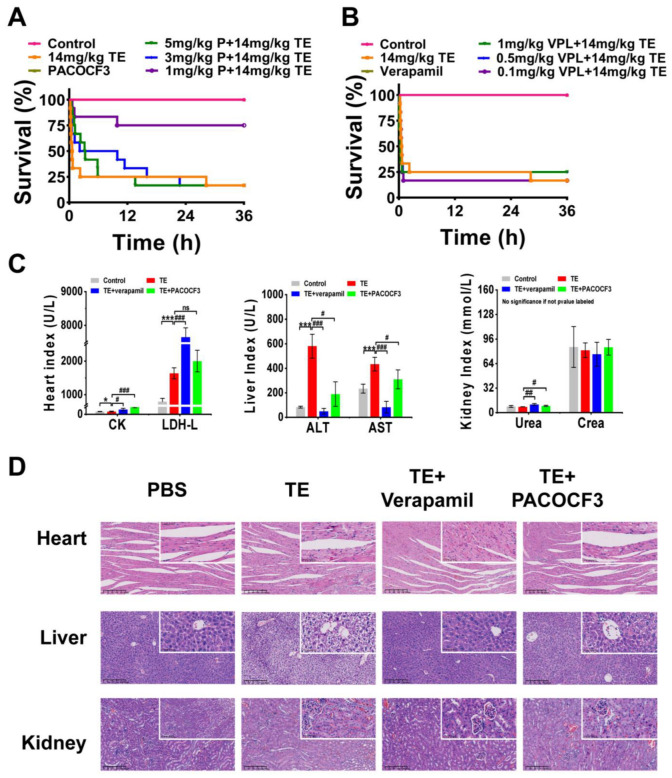
In vivo interventional effects of different inhibitors on TE. (**A**) Lethality analysis of TE interfered with PACOCF3, n = 12. (**B**) Lethality analysis of TE interfered with verapamil, n = 12. (**C**) Evaluation of blood biochemical parameters including creatine kinase (CK) and lactate dehydrogenase (LDH), aspartate aminotransferase (AST), alanine aminotransferase (ALT), creatinine (CREA), and urea nitrogen (UREA) in mice treated with TE (14 mg/kg) or TE combined with PACOCF3 (1 mg/kg) or verapamil (1 mg/kg), n = 3. (**D**) Detailed HE-stained analyses of the heart, liver, and kidneys. 400× magnification, n = 4. * *p* < 0.05, and *** *p* < 0.001 represent the comparison of the group with the control group, and # *p* < 0.05, ## *p* < 0.01, and ### *p* < 0.001 represents the comparison of the group with the TE group, with ns representing no significance.

A mixture of TE and PACOCF3 or verapamil was injected intravenously into ICR mice, and serum and organ samples (heart, liver, kidney) were subsequently harvested and analyzed. At 36 h post-intervention, doses of PACOCF3 ranging from 1–3 mg/kg significantly ameliorated lethality, and a higher dose (5 mg/kg) prolonged their survival time, although it did not improve lethality (Figure 4A). Doses of 0.1–1 mg/kg of Verapamil did not improve lethality associated with TE, nor did they have a positive or negative impact on survival time (Figure 4B). Biochemical tests conducted on mouse serum after treatment with 1 mg/kg of PACOCF3 or Verapamil (4–6 h) demonstrated a significant reduction in ALT and AST levels. However, there was no improvement in other parameters following treatment with either PACOCF3 or Verapamil. Notably, while Verapamil did not enhance survival rates in cases of TE, it unexpectedly showed greater efficacy in reducing ALT and AST levels compared to PACOCF3 (Figure 4C). Histopathologic examination of cardiac, hepatic, and renal tissues after treatment showed an improvement in toxin-induced injury. Specifically, denser cardiac muscle fibers, reduced hemorrhage, more regular liver lobule shape, and reduced edema were observed and were consistent with the blood biochemistry results, suggesting that verapamil was more efficacious than PACOCF3 for hepatic injury (Figure 4D). In addition, in the absence of TE, neither PACOCF3 nor verapamil caused significant damage to the liver or kidneys, and only the heart showed a slight irregularity in the arrangement of cardiomyocytes. Similar to the blood biochemistry, in the absence of TE, PACOCF3 and verapamil both upregulated CK and LDH-L expression, but significantly lowered ALT and AST levels, with no significant changes in UREA and CREA (Figure S4A,B). Thus, we hypothesized PACOCF3 may prevent jellyfish venom-induced lethality and have a hepatoprotective effect, and although verapamil did not ameliorate lethality, it may have also some hepatoprotective activity as a commonly used cardiac drug.

## 3. Discussion

### 3.1. Effects of Different Jellyfish Venom Extraction Methods on Venom Components and Their Activities

The link between fluctuations in the composition or content of toxin proteins and their activity and the influence on subsequent symptoms by different venom extraction methods has been demonstrated in other venomous organisms [55,56]. Although this has been used as a rule for jellyfish [57], our results are consistent. We used a proteomic approach to gain insights into the proteomic differences between *N. nomurai* jellyfish venoms (TE and NV) extracted using two different methods, and the toxicity was evaluated in vitro and in vivo, respectively. The 239 and 225 toxin proteins found in TE and NV were included in 118 protein families. Among the ten highest expression protein families, most of the venom toxins were more abundant in TE than in NV, except for Alpha-1 type I collagen (COL1A1) in NV. COL1A1, as a fibrous collagen, is particularly important for skin, bone, and connective tissues, and is expressed in almost all connective tissues, suggesting that mechanical fragmentation of nematocysts still retains capsid proteins. In addition, COL1A1 has been reported to be involved in the hemorrhagic symptoms of snake venom, promoting the hemorrhagic activity of snake venom zinc-dependent metalloproteinases (SVMPs) [58], a seemingly “non-venomous” protein that has received little attention in jellyfish studies. Other toxins rarely reported in jellyfish venom, such as conotoxin-like proteins, were also identified in our study.

In summary, our results suggest that natural lysis can produce a purer and more toxic venom in jellyfish, and mechanical destruction of the nematocysts can retain a complete venom, including some structural proteins used for venom storage. These provide a new scientific rationale for using TE as a source of jellyfish toxicology, along with increased productivity, which may be helpful for drug development [59]. The extraction of NV may retain a large number of structural proteins, which makes it require higher concentrations to achieve the same cytotoxicity and lethality compared to TE, but this is not absolute. The different extraction methods of TE and NV can result in the loss of some toxin proteins, which may be critical and lethal. Identifying the two different venom extraction methods has dramatically expanded our understanding of the biological properties of jellyfish venom.

Although TE and NV did not exhibit the same cytotoxicity, similar effects at the same cytotoxic concentrations, particularly in the modulation of transcriptome-predicted total protein and phosphorylated expression levels of c-Fos, c-Jun, p65, and p38 genes, were shown in them. As predicted by the transcriptome as well as by the PPI, the mRNA levels of RAW264.7 cellular immunity-related genes (IL6, TNFα, Cxcl10, Ccl2, IL1β, and Cxcl2) were elevated by TE and NV treatments, and NV-induced cytokines were more highly upregulated than those by TE yet were less cytotoxic at the same concentrations. Therefore, the upregulation of these inflammatory factors may not be mainly involved in the venom cytotoxicity. In addition, TE and NV regulate the upregulation of phosphorylated p38, which may play a vital role in the pathogenesis of inflammation by acting on the expression of downstream regulatory factors, such as TNF-α and IL-6 [60].

### 3.2. Toxicological Effects of Venom and Effects on Different Organs

The heart [61,62] and liver [63,64] are the main target organs of jellyfish. A previous study reported that exposure to jellyfish venom resulted in pathologic changes in the heart and liver but not in the kidneys of mice. Consistent with the earlier study [65], mice exposed to TE (12 mg/kg) and NV (23 mg/kg) exhibited structural damage to the heart and liver; for example, cardiac tissue showed structural damage to cardiomyocytes, widening of myocardial interstitium, deformation of liver lobules, hepatocyte degeneration, necrosis, blurring, and the widening of cellular interstitial space, all accompanied by localized inflammatory cellular infiltration, which were consistent with increased activity of CK and LDH-L [66].At the same time, in the case of liver injury, AST and ALT activity enhancement occurred, as these active substances were released into the blood [67], and from the biochemical data, it seems that the TE-treated group was more seriously injured. These results suggest that the venom of *N.nomurai* jellyfish induces more severe heart and liver damage but less kidney damage, but the related toxicity mechanism is unclear. Therefore, further studies are required to investigate the molecular mechanisms of the cardiac and hepatic toxicity of jellyfish venom.

### 3.3. Evaluation of the Ex Vivo and In Vivo Effects of PLA2, MMP, and Calcium Channel Inhibitors

The development of jellyfish venom inhibitors has always been a research hotspot. However, due to the unstable nature of the venom [7], the presence of species, geographic variability [68], and insufficient information about the venom, it has yet to have a specific therapeutic modality, and even serum antibodies have poor efficacy [69]. It has been reported that jellyfish venom has a complex composition, in which PLA2 and MMP are essential components of jellyfish toxins [70]; PLA2 can release free fatty acids and lysophospholipids [71], MMP can induce inflammation [28] and myotoxicity [33], and calcium overload is considered to be the main cause of cardiotoxicity in jellyfish poisoning [72]. As one of the major toxins in terrestrial animals, the PLA2 inhibitor PACOCF3 has been used to inhibit PLA2 activity [73,74]. Furthermore, in jellyfish-related research, MMP inhibitors, like EDTA [51], and calcium channel blockers, including diltiazem [72] and verapamil [75], have been investigated, and have been widely demonstrated to antagonize jellyfish stings, although some of the results may be contradictory [76,77]. This contradiction also exists in our study, where EDTA was reported to be effective in [53] but showed little or no effect on our data. The antagonistic effect of calcium channel inhibitors has been contradictory, and verapamil has been reported to be effective [78] or ineffective [79] in antagonizing jellyfish venom or even exacerbating cases of toxicity [76], which may be related to the species of jellyfish. Thus, confirmation of the stinging species appears necessary when selecting a treatment. In the present study, verapamil did have an excellent antagonistic effect on toxicity at the cellular level, but this antagonistic effect was not observed in an in vivo model. In contrast, PACOCF3 showed strong antagonistic effects both for in vivo and in vitro models of jellyfish toxicity, and this antagonism was accompanied by a decrease in p38 phosphorylation levels in cells. This may suggest that PLA2 may promote MAPK activation, which is associated with jellyfish venom cytotoxicity in RAW264.7, and PLA2 inhibitors may be utilized as potential MAPK inhibitors. This conjecture has been demonstrated in snake venom [80] but remains to be tested in jellyfish

## 4. Materials and Methods

### 4.1. Jellyfish Samples Collection

In October 2023, *N. nomurai* were obtained in the East China Sea, and their tentacles were immediately cut off and stored at −80 °C for nematocyst isolation and venom extraction. No specific permit was required because the jellyfish were not an endangered or protected species. Jellyfish samples were collected from an unprotected or privately owned marine environment.

### 4.2. TE or NV Venom Preparation

TE and NV were prepared separately using widely accepted methods for collecting jellyfish venom. TE was obtained through the natural exsolution of the tentacles, while the venom NV was extracted from the tentacles by mechanically fragmenting the nematocysts [7]. According to the TE collection method described by Li [41], frozen tentacles were placed in a beaker and stirred continuously for 72 h at 4 °C to allow complete autolysis of the tissues. The mixture was filtered through a 200-mesh sieve to obtain the supernatant and centrifuged at 10,000× *g* for 15 min at 4 °C. The supernatant was centrifuged in PBS (0.01%) for 2 h at 0.01%. Then, the dialysate was dialyzed in PBS (0.01 mol/L, pH 7.4) for 12 h. The dialysis venom was labeled as TE. Frozen tentacles were thawed in filtered seawater at 4 °C for autolysis according to the NV collection method described by Li [28], and the seawater was changed every day until most of the visible tissue fragments were dissolved. After autolysis, the mixture was filtered through a 200-mesh sieve to remove tissue fragments. The filtrate was then centrifuged at 3000× *g* for 15 min at 4 °C, the precipitate was collected to obtain the nematocysts, and the nematocysts were washed three times with PBS, then further centrifuged at 10,000× *g* for 15 min at 4 °C. The nematocysts were fragmented using a bead grinder at 400 w. A total of 20 cycles were performed, each consisting of 10 s of sonication and 15 s resting on ice. The venom was then centrifuged at 12,000× *g* to obtain the supernatant and labeled as NV.

### 4.3. Label-Free LC–MS/MS

The TE and NV samples were collected and analyzed by Lianchuan Biological Co. Briefly, the proteins were hydrolyzed by trypsin and quantified. For the LC-MS/MS analysis, the enzymatic products were separated using a Thermo Ultimate 3000 nano ultra-performance liquid chromatography system at a flow rate of 300 nL/min on an Acclaim Pep Map 100 column (2 cm × 75 μm, 3 μm) with a gradient elution at a flow rate of 5 μL/min: 5 to 21% solvent B (97% ACN, pH 9.8) in 38 min, 21.5% to 40% solvent B in 20 min, 40% to 90% solvent B in 2 min, 90% solvent B for 3 min, and 5% solvent B equilibrated for 10 min. The elution peaks were monitored at 214 nm, and fractions were collected every minute. The fractions were combined according to chromatograms of the elution peaks. Ten fractions were obtained and then freeze-dried. Ionization was performed using an Orbitrap Exploris™ 480 mass spectrometer (Thermo Fisher Scientific, San Jose, CA, USA) in DDA (data-dependent acquisition) mode, followed by data-dependent MS/MS scanning. Screened proteins were identified and quantified by MaxQuant software version 2.1.4.0 (https://www.maxquant.org/, accessed on 19 February 2024) under the conditions of false-positive of the peptide-spectrum matches (PSM FDR) < 0.01 and false-positive of protein (protein FDR) < 0.01. The reference database selected for this DDA label-free mass spectrometry raw data (Raw data) was the *N. nomurai* Transcriptome Database. The reference database used for this DDA label-free mass spectrometry raw data (Raw data) was the *N. nomurai* eukaryotic non-coding transcriptome sequencing data (database of 26,066 sequences) (https://www.ncbi.nlm.nih.gov/sra/?term=Stomolophus+meleagris, accessed on 19 February 2024). The *N. nomurai* transcriptome database (database totaling 26,066 sequences) and the Uniprot biotoxin database (database totaling 1,359,673 sequences) were run using the Local Blast tool (e-value of <1.0 × 10^−5^) (https://www.uniprot.org/uniprotkb?query=toxin, accessed on 19 February 2024); then, the identified proteins were named according to the protein names in uniprot, and the families to which the proteins belonged were recorded and categorized according to their family and domain. We annotated the protein sequencing results from the reference N.nomurai eukaryotic non-coding transcriptome database using this record. Toxicity identification only screens results with a sequence alignment consistency exceeding 40%.

### 4.4. Survival Analysis

Male ICR mice with an average weight of 25 ± 2 g were obtained from the Laboratory Animal Center of Naval Medical University. Different concentrations of PBS diluted with TE (0–20 mg/kg, n = 12 of each treatment group) or NV (0–30 mg/kg, n = 12 of each treatment group) [65,81] were injected intravenously into each mouse through the tail vein. Subsequently, the mice were observed continuously for 3 days. Mortality data were collected to calculate the median lethal dose (LD50) for TE intervention experiments. PACOCF3 (0–5 mg/kg) or verapamil (0–1 mg/kg) diluted in PBS were pre-mixed with a dose of 14 mg/kg TE in excess of the LD50, administered through the tail vein, and death time was recorded over a 36 h period. All experiments were conducted using animal welfare principles and approved by the Naval Medical Center of PLA Ethics Committee (NMC2023011).

### 4.5. Histologic Examination and Blood Biochemical Analysis

Four to six hours after the injection of a median lethal dose of TE (12 mg/kg) or NV (23 mg/kg) through the tail vein, orbital blood was withdrawn for haematochemical analysis and then the mice were killed by cervical dislocation. Then, the hearts, livers, and kidneys were dissected and immediately placed in tissue fixative for histological analysis. Briefly, after routine processing, the tissue samples were embedded in paraffin, cut into thin slices, dehydrated in xylene and ethanol, and washed with clean distilled water. Subsequently, the sections were stained using a hematoxylin–eosin dye solution (B1002, Baiqiandu Biotechnology, Wuhan, China).

For blood biochemical analysis, the median lethal dose (LD50) of TE and the inhibitor dose of 1 mg/kg of PACOCF3 or verapamil, as well as 1 mg/kg of PACOCF3 or verapamil premixed with 12 mg/kg of TE, were used for the intervention experiments through the tail vein. Other operations were performed as above. The orbital blood samples were centrifuged at 2000× *g* for 10 min. The resulting supernatant was used as a template for the measurement of various biochemical parameters, including cardiac enzymes (CK, LDH-L), liver function markers (ALT, AST), and renal function markers (CREA, UREA).

### 4.6. Cytotoxicity Assay

RAW264.7 cells were inoculated at 10,000 cells/well in 96-well plates and incubated in DMEM containing 10% fetal bovine serum and 1% penicillin and streptomycin at 37 °C, 5% CO2, and 85% humidity under aseptic conditions for 24 h. Cells were treated with TE and NV at different concentrations (0–100 μg/mL, n = 6) for 2 h. Changes in absorbance were measured at 450 nm by using enzyme labeling instrument (BioTek, Winooski, VT, USA) to measure their absorbance at 450 nm. The wells without venom were used as a negative control and the effect was eliminated by using wells containing only DMEM. The formula for TE was calculated as follows: viability (%) = (ODTE Background)/(ODNC Background) × 100%. Nonlinear fitting was performed by logistic equations on survival curves analyzed using Origin2021 software.

In the inhibitor intervention experiments, cells were treated with a premix of 70% inhibitor dose of TE or NV (IC_70_) and 10–30 μM doses of inhibitors (EDTA, PACOCF3, or verapamil), and the rest of the operations were performed as above. The RAW264.7 cell line was provided by QiDa Biotechnology Co., Ltd. (Shanghai, China).

### 4.7. Transcriptomics Analysis

#### 4.7.1. RNA Extraction and Sequencing

RAW264.7 (2.5 × 10^4^ cells/mL) was inoculated into 12-well plates and treated with TE or NV at a median inhibitory concentration (IC_50_) for 2 h. According to the manufacturer’s guidelines, total RNA was isolated from the cells using an RNeasy RNA Fast Extraction Kit (Feijie RNA fast200, Shanghai, China). Total RNA was analyzed using an enzyme marker (BioTek, Winooski, VT, USA) to assess the concentration and integrity of RNA. RNA samples were analyzed by Lianchuan Biological Co. Briefly, the RNA was quality checked and the sequencing conditions (concentration > 50 ng/μL, RIN value > 7.0, OD260/280 > 1.8, total RNA > 1 μg) were met using oligo(dT) magnetic beads (Dynabeads Oligo (dT), item 25-61005, Thermo Fisher, Waltham, MA, USA). The mRNA with PolyA (polyadenylate) was specifically captured in two rounds of purification. The captured mRNA was fragmented at high temperature, and cDNA was synthesized in the presence of reverse transcriptase (Invitrogen SuperScript™ II Reverse Transcriptase, No. 1896649, CA, USA). E. coli DNA polymerase I (NEB, No. m0209, Ipswich, MA, USA) and RNase H (NEB, No. m0297, Ipswich, MA, USA) were used for two-stranded synthesis to convert these DNA–RNA complex double strands into DNA double strands, and dUTP solution (Thermo Fisher, No. R0133, CA, Waltham, MA, USA) was mixed with the second strand to make the ends of the double-stranded DNA into flat ends. To make the ends of the double-stranded DNA flat, a base was added to each end of the double-stranded DNA to enable it to be ligated to a junction with a T base at the end, and the fragment size was screened and purified using magnetic beads. The second strand was digested with UDG enzyme (NEB, No. m0280, Ipswich, MA, USA), and PCR was used produce a library with a fragment size of 300bp±50bp. Finally, we used Illumina Novaseq™ 6000 (LC Bio Technology Co., Ltd. Hangzhou, China) to perform bipartite sequencing according to the standard operation, and the sequencing mode was PE150.

#### 4.7.2. Differentially Expressed Genes (DEGs) Analysis

The results of comparing genomic and genomic annotation files from RSEM were used to obtain read counts of transcripts that were used for FPKM or TPM transformation to measure gene expression levels. Differentially expressed genes (DEGs) were screened (|log2(FC)| > 1 and *p* < 0.05). Functional enrichment analysis (KEGG pathway enrichment) of DEGs was performed using KOBAS. Based on our previous studies, network diagrams were generated by protein–protein interaction (PPI) network analysis to elucidate the relationships between critical DEGs and to assess the distribution trends of genes in the TE or NV gene sets in gene lists sorted by their phenotypic relevance to the PI3K-Akt pathway and MAPK pathway (GSEA).

### 4.8. Western Blotting

RAW 264.7 (2.5 × 10^4^/mL) was inoculated into cell culture plates, and the cells were treated with median inhibitory concentrations of TE (10.45 μg/mL) or NV (32.34 μg/mL) for 2 h, and then the total proteins were isolated for subsequent experiments. Briefly, total protein lysates from RAW 264.7 cells were prepared using lysis buffer RIPA (Yase, pc101, Shanghai, China). A total of 10–20 μg of protein was separated by 10% SDS-PAGE (Epizyme, PG212, Shanghai, China) by adding phosphatase inhibitor (Epizyme, GRF102, Shanghai, China) and protease inhibitor (Epizyme, GRF101, Shanghai, China) to the lysate according to the manufacturer’s instructions, and then transferred onto a nitrocellulose membrane (Cytiva, 10600002, Washington, DC, USA). The membranes were enclosed in a rapid containment solution (Aase Bio, China) and incubated with the first specific rabbit monoclonal antibodies c-Fos (1:1000), p-c-Fos (1:1000), c-Jun (1:1000), p-c-Jun (1:1000), p38 (1:1000), p-p38 (1:1000), p65 (1:1000), p-p65 (1:1000), and β-Actin (1:10,000), which were incubated at 4 °C for 12–16 h. After washing with TBST, each membrane was incubated with the second anti-rabbit IgG goat polyclonal antibody for 1 h at room temperature, and the protein bands were detected by chemiluminescence (BLT). Except for p-c-Fos (ImmunoWay, YP0442, Beijing, China) and p-p65 (CST, 3033T, Danvers, MA, USA), all other antibodies were obtained from HuaBio, Hangzhou, China (Antibodies c-Fos: ET1701-95; c-Jun: ET1608-3; p-c-Jun: ET1608-4; p38: ET1702-65; p-p38: ER2001-52; p65: ET1603-12; β-Actin: ET1702-67; anti-rabbit IgG goat polyclonal antibody: HA1001).

### 4.9. RT-qPCR

Total RNA was extracted similarly to in Section 4.7.1. Then, RNA was reversed to cDNA using the PrimeScript RT kit (AG Bio, Hunan, China), and the reverse transcription procedure was as follows: 37 °C for 2 min, 55 °C for 15 min, 85 °C for 5 min, and then maintained at 4 °C. The qpcr procedure was as follows: 94 °C for 5 min for pre-mutability, 94 °C for 20 s, 60 °C for 20 s, and 72 °C for 30 s, for amplification 40 times. The 2^−ΔΔCt^ method was used to calculate the gene expression using β-Actin [82] as the reference gene. The primers for all genes are listed in Table S1.

### 4.10. Metalloproteinase Activity

The metalloproteinase activity of TE was assayed by the azo-casein method. Different concentrations of TE or NV (0–100 μg/mL, n = 4) were added to a solution (Tris-HCl, PBS, NaCl, CaCl2, and Azocasein (Sigma, A2765, City of Saint Louis, MO, USA)) at pH 8.8, and the mixture was incubated in a water bath at 37 °C for 90 min. After incubation, 200 μL of 0.5 M trichloroacetic acid (TCA) (Ronoen, China) was added to each sample, and the mixture was kept at room temperature for 30 min. The samples were then centrifuged at 10,000× *g* for 10 min. 100 μL of supernatant was collected from each sample and mixed with 100 μL of 0.5 M NaOH (Amresco, 1310-73-2, Washington, DC, USA). The absorbance of these mixtures was subsequently measured at 450 nm.

### 4.11. PLA2 Activity

Different concentrations of TE or NV (0–100 μg/mL, n = 4) were prepared and then diluted to 250 μL with 50 mM Tris-HCl, 5 mM CaCl_2_, and 100 mM NaCl, pH 8.0. Then, 25 μL, 1 mg/mL of 4-nitro-3-octanoyloxybenzoic acid (NOBA) was added; meanwhile, 50 mM Tris-HCl, 5 mM CaCl_2_, 100 mM NaCl, pH 8.0, and PBS were used as controls. Subsequently, the plates were incubated at 37 °C for 1 h, and the absorbance was measured at 405 nm.

### 4.12. Statistical Analysis

All data were expressed as mean ± standard deviation (SD), and were analyzed by one-way ANOVA with * *p* < 0.05, ** *p* < 0.01, and *** *p* < 0.001 representing the comparison of the group with the congrol, and # *p* representing the comparison of the group with the TE group. All statistical analyses were performed using SPSS software 26.0 (SPSS Inc., Chicago, IL, USA). Differences between groups with *p* < 0.05 were considered significant.

## 5. Conclusions

The present study demonstrated that different extraction methods result in variations in the composition toxicity and effects of jellyfish venom, and different inhibitors may exhibit different antagonistic activities in vitro and in vivo. Comprehensive proteomic, transcriptomic, and biochemical analyses revealed detailed differences in toxicity and effects, including inhibitor interventions. In conclusion, the natural lysis method may result in purer venom, and the mechanical fragmentation method may result in more intact venom glands, which were largely indistinguishable from each other in modulating ex vivo and ex vivo toxicity effects, with TE being more toxic and causing more tissue damage, and NV being able to cause upregulation of inflammatory factors at a higher level. In addition, neither verapamil nor EDTA was considered as a suitable inhibitor, and PACOCF3 showed good antagonistic effects against jellyfish poisoning both in vitro and in vivo. Therefore, the present study may provide new insights for the establishment of a scientific and economical source of jellyfish sting venom and the development of therapeutic methods for jellyfish poisoning.

## Data Availability

The authors declare that all relevant data supporting the findings of this study are available within the article and its Supplementary Material files. The data related to the histology research will be uploaded to the NCBI database after the article is accepted.

## Supplementary Materials

The following supporting information can be downloaded at: https://www.mdpi.com/article/10.3390/md22080362/s1. Supplementary figures: Figure S1: Comparison of toxins and toxicity evaluation of TE and NV. Figure S2: TE vs. NV transcriptome overview and validation. Figure S3: PACOCF3 and verapamil antagonized the toxicity of TE or NV in vitro. Figure S4: In vivo intervention effects of inhibitors on TE and their comparison. Supplementary table: Table S1: PCR primer sequences.
