# Supplementary material for: Comparative Analysis of Tentacle Extract and Nematocyst Venom: Toxicity, Mechanism, and Potential Intervention in the Giant Jellyfish Nemopilema nomurai
Source: Mar Drugs. 2024 Aug 9;22(8):362. doi: 10.3390/md22080362 (PMC11355847; doi:10.3390/md22080362)
Supplement: Supplementary file 1 [file marinedrugs-22-00362-s001.zip › marinedrugs-3105259-supplementary.pdf]

Supplementary

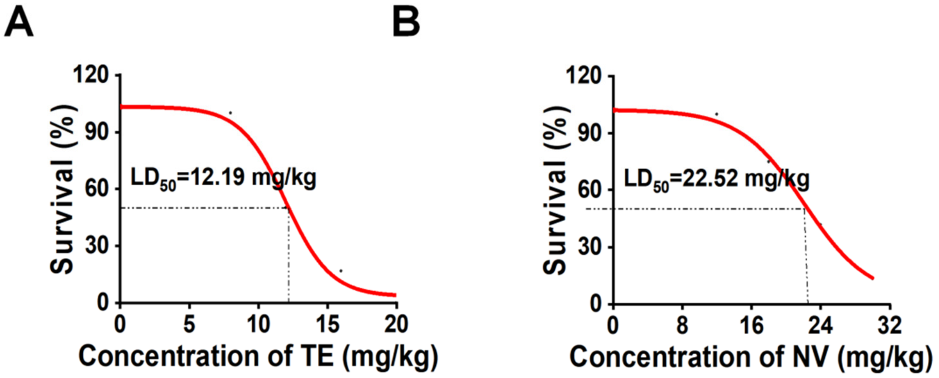

Figure S1. Comparison of toxins and toxicity evaluation of TE and NV. (A) Determination of median lethal dose (LD50) in ICR mice after TE injection, n=12. (B) Determination of median lethal dose (LD50) in ICR mice after NV injection, n=12.

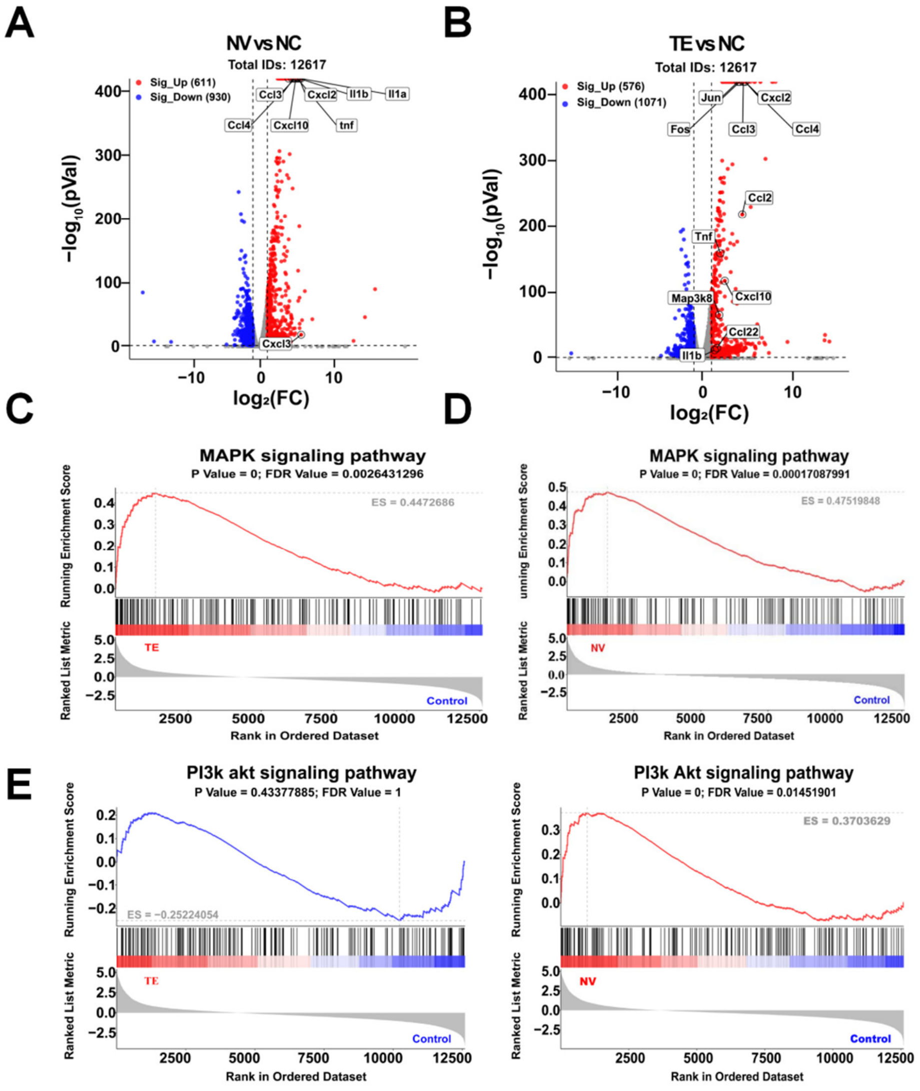

Figure S2. TE vs. NV transcriptome overview and validation. (A-B) Volcano plot of genes up-regulated by TE vs. NV treatment in RAW264.7 cells compared to PBS treatment, n=4. (C-D) GSEA analysis of MAPK in the up-regulated pathway by TE vs. NV treatment in RAW264.7 cells.

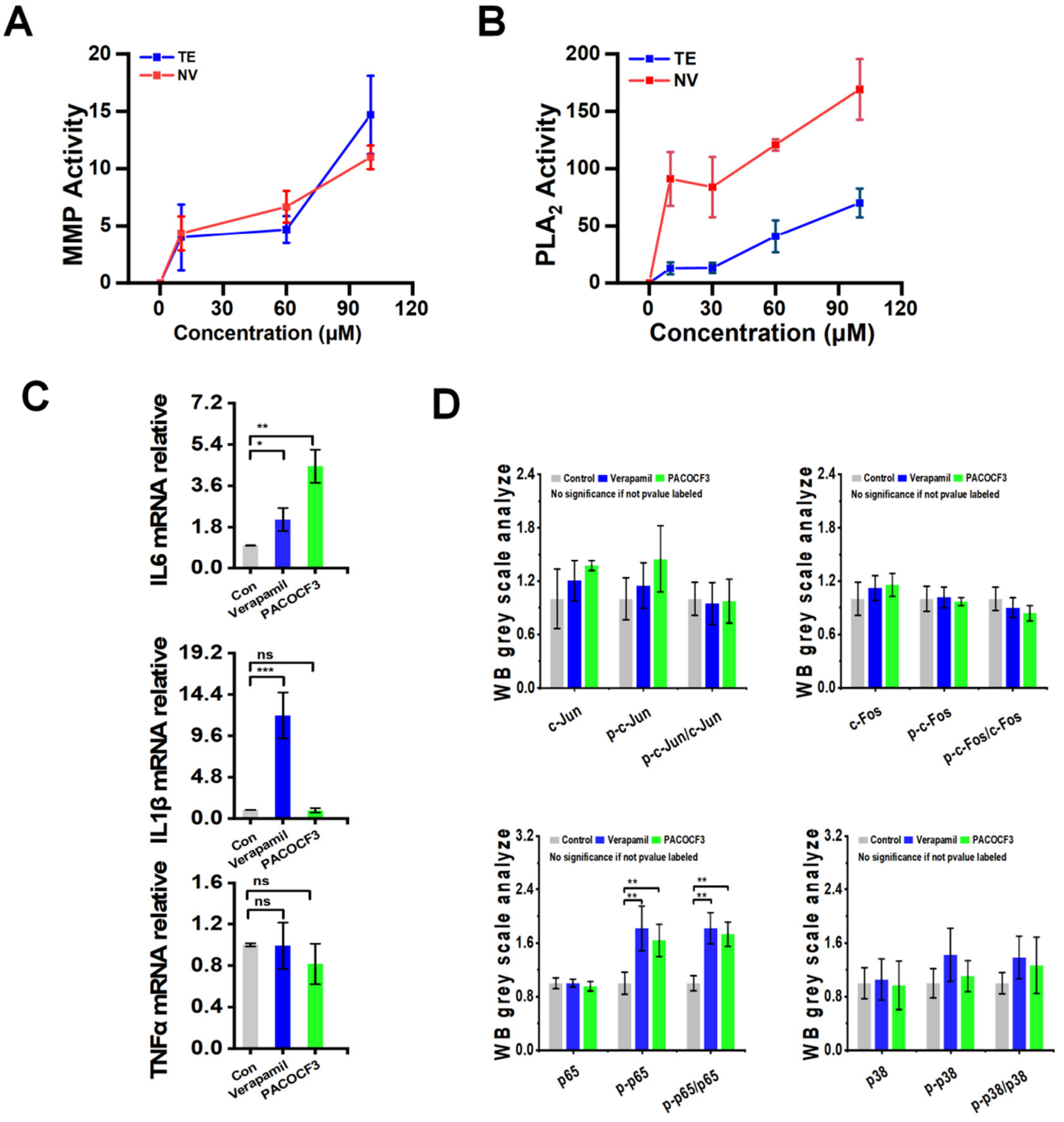

Figure S3. PACOCF3 and Verapamil antagonized the toxicity of TE or NV in vitro. (A) Metalloproteinase activity of TE and NV. (B) Phospholipase A2 activity of TE and NV. (C) Relative gene expression levels of three inflammatory factors, IL-6, TNF-α and IL1β in RAW264.7 cells treated by PACOCF3 (20 μM) or Verapamil (20 μM) .n = 3 (D) Relative gene expression levels of three inflammatory factors, IL-6, TNF-α, and IL1β, in RAW264.7 cells treated by PACOCF3 (20 μM) or Verapamil (20 μM). n = 3. (D) Western blotting analysis of c-Fos, c-Jun, p65, p38 phosphorylated and non-phosphorylated protein expression levels in RAW264.7 cells treated by PACOCF3 (20 μM) or Verapamil (20 μM), n = 4. $^{*}p < 0.05$, $^{**}p < 0.01$, and $^{***}p < 0.001$ representing the comparison of the group with Control, ns representing no significance.

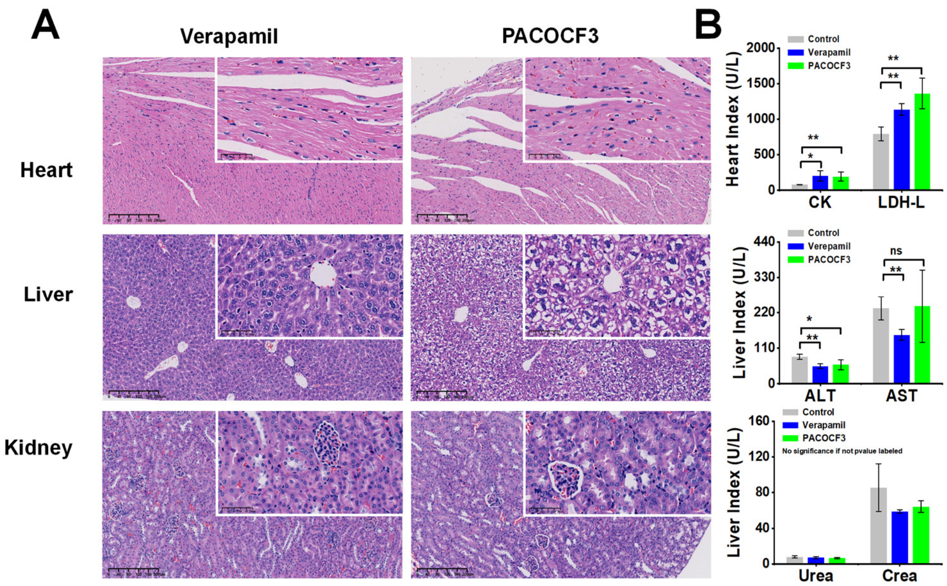

Figure S4. In vivo intervention effects of inhibitors on TE and their comparison. (A) Detailed analyses of heart, liver, and kidney under 400 × magnification, n = 4. (B) Evaluation of blood biochemical indices including creatine kinase (CK), lactate dehydrogenase (LDH), aspartate aminotransferase (AST), alanine aminotransferase (ALT), creatinine (CREA), urea nitrogen (UREA) in mice treated with PACOCF3 (1 mg/kg) or Verapamil (1 mg/kg), n = 3. $^{*}p < 0.05$, $^{**}p < 0.01$, and $^{***}p < 0.001$ representing the comparison of the group with Control, ns representing no significance.

Table S1. PCR Primer Sequences

**Prim name**	**Sequences (5’ > 3’)**
TNFα-F	CGCTGAGGTCAATCTGC
TNFα-R	GGCTGGGTAGAGAATGGA
CXCL10-F	AGCCGTGGTCACATCAG
CXCL10-R	ATCCCAGCCACTTGAGC
IL6-F	ACAGAAGGAGTGGCTAAGGA
IL6-R	AGGCATAACGCACTAGGTTT
IL1β-F	TCATTGTGGCTGTGGAGAAG
IL1β-R	TCATCTCGGAGCCTGTAGTG
CXCL2-F	ACCAACCACCAGGCTACAGG
CXCL2-R	GCTTCAGGGTCAAGGCAAAC
CCL2-F	GCATCTGCCCTAAGGTCTTCA
CCL2-R	GTTCACTGTCACACTGGTCACTCC
MM-ACTINB-F	GTGCTATGTTGCTCTAGACTTCG
MM-ACTINB-R	ATGCCACAGGATTCCATACC
